# Effect of Repeated Moist Heat Sterilization on Titanium Implant–Abutment Interface—An *In Vitro* Study

**DOI:** 10.1055/s-0043-1776314

**Published:** 2024-01-10

**Authors:** Mohamed S.M. Morsy, Ali Abdel-Halim Abdel-Azim Hassan, Hamed A. Alshawkani, Khurshid A. Mattoo, Ankita Mathur, Luca Fiorillo

**Affiliations:** 1 Department of Prosthetic Dental Sciences, College of Dentistry, Jazan University, Jazan, KSA; 2 Department of Maxillofacial Surgery and Diagnostic Sciences, College of Dentistry, Jazan University, Jazan, KSA; 3 Department of Restorative Dental Science, College of Dentistry, Jazan University, Jazan, KSA; 4 Department of Periodontology, Dr. D.Y. Patil Dental College and Hospital, Dr. D.Y. Patil Vidyapeeth, Pimpri, Pune, Maharashtra, India; 5 Department of Biomedical and Dental Sciences, Morphological and Functional Images, University of Messina, Messina, Italy; 6 Multidisciplinary Department of Medical-Surgical and Dental Specialties, Second University of Naples, Naples, Italy

**Keywords:** titanium abutments, implant–abutment interface, sterilization, marginal gap, surface roughness, dental implant

## Abstract

**Objectives** Sterilization eliminates microbial viability by decreasing the biological load, but likewise have the ability to deteriorate the mechanical properties of an implant material. This study intended to evaluate the effect of repeated moist heat sterilization on implant–abutment interface using two different implant systems.

**Materials and Methods** Forty screw-retained titanium implant–abutment combinations (fixture 3.5 ×10 mm, abutment 2 mm diameter), twenty each from Genesis (Aktiv Implant Systems, United States) and Bredent (SKY, Germany), were divided into four different groups ( *n* = 10) and placed in a computer-aided diagnostic model. The abutments from each group were exposed to first and second autoclave cycle (121°C for 30 minutes), connected back to the fixture and analyzed under scanning electron microscope for marginal gap and surface roughness.

**Results** Genesis group showed higher marginal gaps on both sides (buccal/mesial [2.8 ± 0.47]; lingual/distal [2.8 ± 0.33]), while Bredent implant–abutment system (IAS) did not show any changes in marginal gaps after autoclaving. Differences within and between the group were found to be statistically significant. Surface roughness for Genesis (243.7 ± 70.30) and Bredent groups (528.9 ± 213.19) was highest at second autoclave, with Bredent implant–abutment showing higher values for surface roughness than Genesis IAS.

**Conclusion** Marginal vertical gap increased with autoclaving for Genesis IAS, while Bredent implant abutments were more stable. Surface roughness increases with autoclaving for both Genesis and Bredent group of IAS.

## Introduction

Biological advance of osseointegration with the titanium surface with the consequent introduction of Osseo integrated implants has been considered as one of the most significant revolutions in the field of dentistry in recent times. 1 2 In 1965, Brånemark after inserting a titanium implant, observed bone adherence with titanium as “osseointegration,” which later evolved as a separate and totally new field in the world of dentistry. 3 Among the vast array of treatments offered in dentistry, implant therapy stands out for its promising results in multiple prosthodontic applications like single tooth replacement, 4 implant supported fixed and removable partial dentures, fully bone anchored complete denture prosthesis, and implant overdentures. 5 Its advantages also have influenced other treatment modalities in planning their treatments, an example being the implant placed within the bone while other treatments are carried on. 6 This is based on the fact that osseointegration develops over a period of 4 to 6 months depending upon the location and type of available bone.

Titanium alloy as implant material occupies the superior most position that is related mainly to its osseous biocompatibility. The most frequently used dental implant system is marketed as two-piece implant systems: implant body and abutment. Abutment being the connecting component between submerged implant fixture within the bone and primarily assists in retaining and supporting the overlying dental prosthesis. 7 With a variety of options in the materials available for implant abutment, titanium abutments are the most used abutments due to its high survival rate. 8 The connection between implant abutment forms a critical interface between the implant body and the abutment that can determine the clinical outcome of any implant system irrespective of the material used. This interface has been considered an important landmark for microbial colonization. It presents as a microgap with both vertical and horizontal discrepancies present between the overlying abutment and the underlying implant surface.

Despite high success rates of osseointegrated implant (90–10 years), 9 mechanical problems can occur in the implant–abutment system (IAS), leading to failure. These can lead to microbial proliferation that causes inflammation around the implant soft tissues leading to peri-implantitis. 10 Hence, a high precision fits between the implant body and the abutment is paramount for implant body–abutment connection stability. Friction between the two similar surface components can lead to the removal of the surface protection layer and alter the surface properties of the metal. Recent studies have reported a high quantity of titanium elements in biofilms derived from the submucosa around the implant–abutment interface (IAI) that had resulted in peri-implantitis. 11 Such titanium by-products have been attributed to material degradation by a method categorized as tribocorrosion. 8 Alternately, tribocorrosion can also be due to other causes that involve implant fixture and abutment use.

Abutment sterilization after laboratory or clinical use is common practice in implant dentistry. Sterilization procedures eliminate viable microorganisms thus reducing the biological load after sterilization. 12 Repeated sterilizations, however, can lead to deterioration of mechanical properties required for the accurate clinical fit of the abutment and thus may need to be corrected through indirect procedures whether in laboratory or outside oral cavity. Repeated adjustments also have been found to alter the torquing levels of implant–abutment assembly. 13 Genesis and Bredent IAS boast of having superior biomechanical properties like form fit, passive fit, making precise impressions and made from high-quality materials with extensive literature supporting the claims. This study was therefore intended to evaluate the effect of repeated moist heat sterilization on IAI using two different implant systems, that is, Genesis and Bredent IAS. We hypothesize that multiple autoclaving might alter surface properties that in turn alter biocompatibility of the abutment. Alternately, the null hypothesis would state that there will be no surface changes in the implant abutment.

## Materials and Methods

### Sample Selection and Grouping

Two different IAS were used, that is, Genesis (Aktiv, Inc, United States) and Bredent (SKY, Senden, Germany) implant abutments. Forty screw-retained titanium implant abutments were included out of which 20 abutments belonged to the Genesis group and twenty belonged to the Bredent group. Sample size was calculated using G*Power 3.1.4.2. Four different groups (Gp 1—presterilized genesis; Gp 2—presterilized Bredent; Gp 3—first cycle autoclaved genesis [3a] and Bredent [3b]; Gp 4—second cycle autoclaved genesis [4a] and Bredent [4b]) were divided, each comprising of 10 abutments each ( *n* = 10). These abutments were screwed to their respective implant fixtures. Four implant fixtures were included in this study of the same dimension, that is, 3.5 ×10 mm, for each of the four groups. A straight universal abutment for respective groups was standardized in terms of diameter and platform type (2 mm diameter and regular platform). Both groups received their recommended respective implant abutments while following each manufacturers guidelines for placement and removal.

### Sample Preparation (Experimental Procedures)

#### Preparation of Computer-Aided Diagnostic Model

A three-dimensional (3D) model replicating the mandibular arch in the premolar and molar area was created by a computer-aided diagnostic (CAD) software Pixologic Zbrush 2.0 (Pixologic, California, United States) using cone-beam computed tomography generated data report of the edentulous mandibular arch. This model was designed virtually and curated to form a standard mandibular arch form. It was further sectioned virtually in the area of the first molar to form model blocks of approximately 1 ×1 cm to receive an individual implant. The CAD model was designed and manufactured according to this model's specifications. Four such blocks were designed in total to receive four implants from four different groups. These sections of the mandibular arch form were then retrieved and fabricated using 3D printing.

#### Virtual Designing and Printing of a Standard Reference Model of the Sectional Arch Form of the Edentulous Mandible

Stereolithography or “SLA” is an additive manufacturing process that, in its most common form, works by focusing an ultraviolet laser onto photopolymer resin. The CAD design obtained above was then derived in stereolithography format (stl) and transferred to 3D printer form 2 Basic (Form Laboratories, USA) to print a standard study model using SLA technology with 100% infill density. 3D models of the sectional mandibular arch were printed. A total of four such blocks were fabricated using 3D printing, two for each group.

#### Sequential Implant Drilling and Marking the Model

The implant sites were marked after locating the resin model's exact center using perpendiculars drawn from buccal/lingual and mesial/distal sides. For each implant system (Gp 1 and Gp 2), their respective implant drilling kits were used. A pilot drill (2.3/2.0 mm, Short, PD2.3S) was initially used up to 8 mm of height, followed by sequential larger surgical drills (2.8/2.3mm, Short SD 2.8S) and finally a surgical drill (3.4/2.8mm, Short, SD 3.4S) to achieve a total of 10 mm of osteotomy height within each model in each group. For Gp 2 (Bredent SKY), implants were placed in the resin model using sequential drilling [Pilot drill (SKYDP08) followed by a surgical drill (SKYD3435)]. Both implants were placed manually using respective implant drivers (Gp 1—[2.4 mm, Short, IDS]; Gp 2—[SKY-STK6]). Implants for both the systems were placed in the model with torque ratchet with a final torque achieved as 35N.

After the implants were screwed on their respective models, four areas were defined on the mounted implant model: buccal, mesial, palatal, and distal by marking boundaries along the model using four dots/notch onto the model surface for easy scanning. These notches were made using a fine diamond round bur and turbine under a stereomicroscope. All these four dots were color-coded using permanent color markers: buccal—red, mesial—green, lingual—blue, distal—yellow to better recognize the side and ease of scanning.

#### Intervention (Steam Autoclave)

A single steam autoclave (Tuttnauer Model T-Max-10; SN-14060015) was used to sterilize the samples of all groups. A total of 10 samples each of Genesis (Aktiv) Straight Universal abutments of 2 mm dimension and Bredent (SKY) Straight Universal abutments of 2 mm dimension formed the third and fourth study groups. These abutments were prepared for the first cycle of an autoclave by wrapping and packing using Oro Sterilization Reels—100mm heat sealing flat reel. The autoclave temperature ranged between 105 and 138 degrees at various times of sterilization (initial heating, sterilization, drying), while a standard temperature of 121°C for at least 30 minutes by using saturated steam under at least 15 psi of pressure was maintained to ensure uniform sterilization protocol. After the first autoclave cycle, abutments from the third and fourth group were screwed on their respective implant systems and analyzed under a scanning electron microscope (SEM). The second autoclave cycle was performed on the same groups of implants from third group and fourth group and then again analyzed for any changes under a SEM.

#### Measures, Data Collection, Evaluation, and Analysis

The respective abutment in each group was screwed onto the dental implant with respective torque ratchets (Torque 35 Ncm; Genesis Aktiv, Bredent SKY) following the manufacturers' guidelines. With Gp 1 and Gp 2 forming the control groups for Gp 3 (a, b) and Gp 4 (a, b) respectively, each specimen was scanned using a SEM (Vega3 TESCAN). The vertical gap and surface changes on the abutment were then analyzed. Each specimen was gold-sputtered (K650 sputter coater, Quorum Technologies), and SEM was used to image the implant–abutment gap at the marginal interface. 14 Implant–abutment connection was held in parallel to the SEM machine's detector to ensure the appropriate positioning of the sample and appropriate vertical gap analysis and alternate two aspects (buccal and mesial) and (lingual and distal) were scanned. For each specimen facet, the marginal fit was imaged by obtaining a scan in a perpendicular direction using 1000× magnification at a set angle. For each scan, three vertical marginal gap measurements using software (Quartz PCI Image analyzer, version 5.5, Quartz Imaging Corporation) were taken. From these three measurements, a mean value for each side was calculated. Thus, for every two sides of each specimen, two measurements were recorded. For surface analysis software, Vega3 TESCAN surface detection software checked the surface roughness. For specimens in Gp 3 (a, b) and Gp 4(a, b), after each autoclaving cycle (cycle 1 and 2) the specimens were scanned using SEM with similar procedure as that of their respective control groups.

#### Digital Examination of Scanned Images

**Fig. 1:**
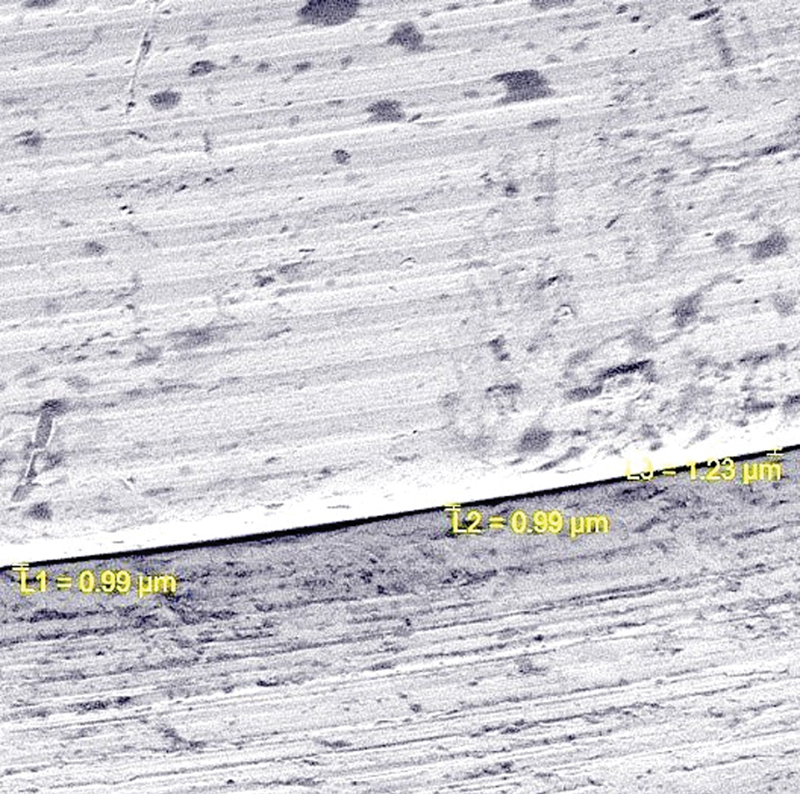
Vertical marginal gap measurement using Scale 2.3, Quartz PCI, version 5.5, Quartz Imaging Corporation.

**Fig. 2:**
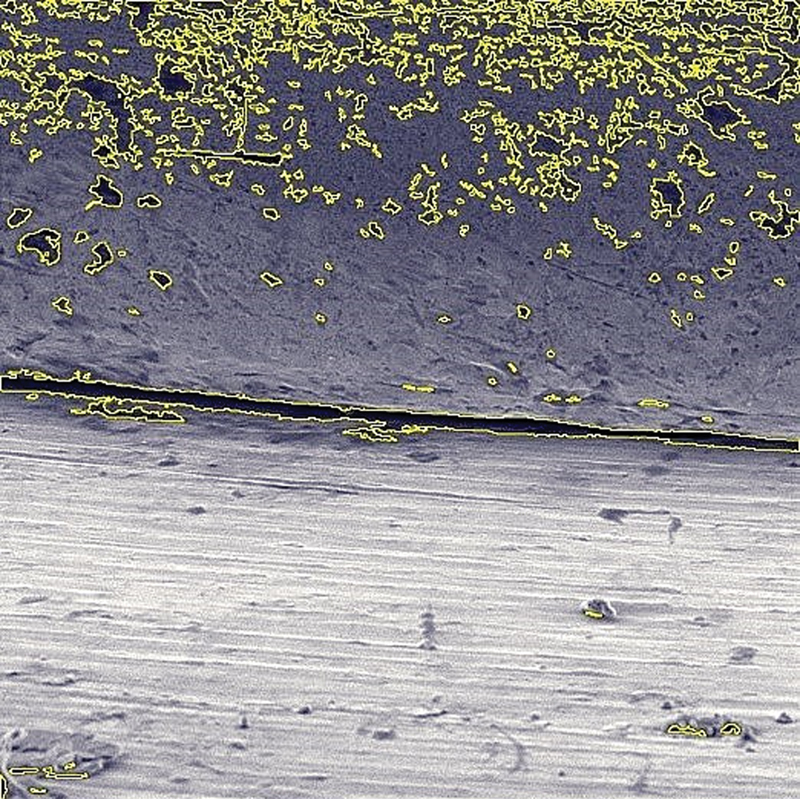
Abutment surface roughness detection using inbuilt Vega3 TESCAN Surface detection.

Once all the samples were scanned, they were collected and assembled in Joint Photographic Experts Group format. All the images were evaluated for any error. Once all the images were verified to be correct, they were further divided into appropriate respective sections that belonged to their respective groups. All four groups were measured separately for the perpendicular marginal difference and surface roughness. Vertical marginal measurements were made using a numeric measuring tool in the software Scale 2.3, Quartz PCI (Version 5.5, Quartz Imaging Corporation, Vancouver, BC, Canada). Vertical measurement was done at three different predetermined sites and the average value of these three measurements was considered the average vertical marginal gap ( Fig. 1 ). Surface roughness measurements were made with inbuilt Vega3 TESCAN, Surface detection software (basic operation parameters: magnification [50 000 × ], beam energy [5–30 keV], scan speed [3 μs/pixel], beam current [1 pА–40 nA]). The surface of the respective abutment was analyzed digitally using the built-in software, which works on pixel capture ( Fig. 2 ).

### Statistical Analysis

The data was entered and analyzed using Statistical Package for Social Sciences (SPSS 24.0; SPSS Inc., Chicago, Illinois, United States) for Windows 11 (Pro, Microsoft corporation). Mean values with standard deviation were calculated for each group. Distribution of data for normality was determined by Kolmogorov–Smirnov test. Differences within the group from baseline to intervention were determined using one-way analysis of variance test with Bonferroni's post hoc correction. Differences between the respective groups at various time intervals were calculated using an unpaired “t” test. The differences between the groups were considered to be significant statistically if the probability of *p* -value was less than or equal to 0.05.

## Results

**Fig. 3:**
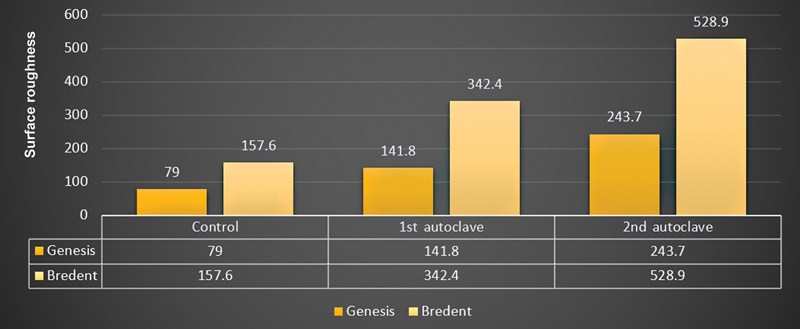
Comparison of mean values of surface roughness in both groups.

The results obtained for marginal gaps on buccal and mesial sides are presented in Table 1 . The results show that before sterilization (control Gp), the lowest mean marginal gap between the two IAS was in Bredent implant system (1.3 ± 0.39) as compared with genesis implant system (1.4 ± 0.21); however, the differences were not significant. After first autoclaving, the mean marginal gap increase was higher for genesis implant system (1.9 ± 0.34) with the difference in the change at first autoclave being significantly different ( *p* = 0.02). At second autoclave, the mean marginal gap of the genesis implant system continued to increase (2.8 ± 0.47), almost becoming double from the control values, while the marginal gap in the Bredent implant system remaining almost same (1.2 ± 0.20). The differences between the two groups at second autoclaving were also found to be highly significant ( *p* ≤ 0.001). Within each implant system, the results show that the changes in mean marginal gap values for control to second autoclaving were higher for genesis implant systems with differences being statistically significant ( *p* = 0.03), while the Bredent implant system did not show any changes in mean marginal gaps after second autoclave cycle. Table 2 presents the mean marginal gaps on the lingual and distal sides of both IAS at various intervals. The results show that there were more differences in mean marginal gap in the genesis implant system from the control (1.4 ± 0.19) to second autoclave (2.8 ± 0.33) as compared with the Bredent implant system. Differences between the two implant system at first and second autoclaving were highly significantly different ( *p* ≤ 0.0001). Within the group, there was no change observed in Bredent implant system ( *p* = 0.4), while there were significant differences in the genesis implant system ( *p* = 0.02). The surface roughness values observed before autoclaving (control) were found to be more in Bredent implant system (157.6 ± 48.13) as compared with genesis implant system (79.0 ± 40.99) that increased in both systems at first autoclaving (genesis −141.8 ± 38.17; Bredent - 342.4 ± 97.46) and second autoclaving cycle (genesis −243.7 ± 70.30; Bredent - 528.9 ± 213.19; Table 3 ). The differences in mean surface roughness values were found to be statistically highly significant for both implant systems except the differences between the two before sterilization (control groups). Bredent IAS reported to have higher surface roughness values as compared with genesis implant system at all-time intervals ( Fig. 3 ).

**Table 1:** Comparison of marginal gaps on buccal and mesial sides in both groups

Marginal gaps on buccal and mesial	Genesis M ± SD	Bredent M ± SD	*t* -Value	*p* -Value
Control ( *n* = 10)	1.4 ± 0.21	1.3 ± 0.39	0.37	0.7
First autoclave	1.9 ± 0.34	1.4 ± 0.26	3.5	0.02 a
Second autoclave	2.8 ± 0.47	1.2 ± 0.20	9.8	0.0001 a
F-Value	8.4	0.90		
*p* -Value	0.03 a	1.0		

▪ Abbreviations: Gp, group; M, mean; *n* , number; SD, standard deviation.

Levels of significance: NS (not significant) = *p* ≥ 0.05.

a Significant = *p* ≤ 0.05.

**Table 2:** Comparison of marginal gaps on lingual and distal sides in both groups

Marginal gaps on lingual and distal	Genesis M ± SD	Bredent M ± SD	*t* -Value	*p* -Value
Control	1.4 ± 0.19	1.4 ± 0.40	0.30	0.7
First autoclave	2.0 ± 0.39	1.3 ± 0.22	4.72	0.0001 a
Second autoclave	2.8 ± 0.33	1.2 ± 0.14	13.79	0.0001 a
*F* -Value	6.7	1.27		
*p* -Value	0.02 a	0.4		

Abbreviations: Gp, group; M, mean; *n* , number; SD, standard deviation.

Levels of significance: NS (not significant) = *p* ≥ 0.05.

a Significant = *p* ≤ 0.05.

**Table 3:** Comparison of surface roughness in both groups

Surface roughness	Genesis M ± SD	Bredent M ± SD	*t* -Value	*p* -Value
Control	79.0 ± 40.99	157.6 ± 48.13	−3.90	0.121
First autoclave	141.8 ± 38.17	342.4 ± 97.46	−6.06	0.0001 a
Second autoclave	243.7 ± 70.30	528.9 ± 213.19	−4.01	0.001 a
F-Value	28.69	18.94		
*p* -Value	0.0001 a	0.002 a		

Abbreviations: Gp, group; M, mean; *n* , number; SD, standard deviation.

Levels of significance: NS (not significant) = *p* ≥ 0.05.

a Significant = *p* ≤ 0.05.

## Discussion

This study was conducted to evaluate whether repeated adjustments of abutment that lead to repeated sterilization bring about changes in implant–abutment connection in terms of the vertical marginal gap and surface roughness, both of which could be a potential cause of bacterial colonization in etiology of peri-implantitis. The implant systems chosen for the study were the most commonly commercially available systems. The main findings of the study were that autoclaving did have a significant effect on marginal gaps (all surfaces) in the Genesis implant system, while having little or no effect on Bredent implant system. The surface roughness increased for both implant systems with Bredent showing more alteration in surface than genesis IAS.

Long-term clinical success of the implant supported restorations depends on multiple factors, with the surface properties of the dental implant being one of utmost significance. Although surgical factors play significant role in early osseointegration, factors related to prosthesis have a major role in the survival of implants. 15 Despite technical and scientific developments in dental implant systems, the IAI still continues to be a major challenge. 16 The marginal gap present at the IAI ideally should not exist as it can have a negative influence on both prosthesis and the surrounding soft tissues by encouraging bacterial growth. The presence of bacteria can contaminate the internal portion of osseointegrated implants that may result in contamination of the implant fixture or pillar. 17 Contamination may also occur by the transmission of microorganisms from the oral medium after the prosthetic pillar placement through the gap present at IAI. The results of our study showed that marginal gaps did exist in both implant systems. These findings are in agreement with the study done by Broggini et al who compared two-piece abutments with one-piece abutment and found more accumulation of inflammatory peri-implant cells with two-piece implants. 18 Authors 19 20 21 in their respective studies affirmed the need of accurate assembling implant components that impart precise prosthesis fit that is necessary for implant survival on the long run through bone preservation around the implant.

The results also show that the first autoclaving of the abutment increased the marginal gap in Genesis IAS that was significant from baseline, while Bredent IAS had an increase in marginal gap but it was not considered to be significant. At second autoclaving, the marginal gap in the genesis group was 2.8 ± 0.47, which was twice than baseline. Sterilization does affect the mechanical properties of the implant surface, especially the one made of titanium. Sutton and Saunders 22 asserted that sterilization in the autoclave is responsible for alterations in the mechanical properties leading to plastic deformation in steel materials when submitted to high levels of stress. They also stated that, due to the lower rate of dissolution of the titanium oxide, this metal shows a passive state and is, therefore, less prone to corrosion. In contrary to stainless steel, both corrosion and temperature contribute to the precipitation of carbonates in its microstructure leading to the structural weakness that does not happen with titanium due to the passive state of titanium oxide. 23 Commercially available pure titanium shows mechanical characteristics such as ductility inferior to alloys. Regarding temperature, this metal shows a hexagonal structure, while at room temperature—the so-called α-phase. 8 The first structural modification occurs at 882°C when the metal shows a cubic structure of a centered body called β-phase. In this last phase, the titanium is hard and fragile, whereas in the α-phase it is ductile and resistant. The mechanical alteration in titanium occurs at high temperatures, around 882°C, that are higher when compared with those in the sterilization process. Despite time and temperature, there is another factor to take into consideration during sterilization that is the humidity and coefficient of thermal expansion at different temperatures of autoclaving. This later can contaminate the layer of titanium oxide with ions F, Fe, Mg, Si, Cl, N, H, and O. 24 Autoclaving process altered the microgap in one of the implant systems (Genesis) used in the current study that may be a result of heat deformation, which can be studied further. It must be also noted that the clinical technique used while placing the implant abutment on the fixture also plays a significant role in the amount of the marginal gap present at this interface, 25 which have prompted different new clinical techniques in the scientific literature.

The practice of reusing implant components has been evaluated previously with respect to the ability to provide a sterile component that provides an economical advantage to the patient or the clinician. 26 Although many clinicians suggest this practice is performed for the patients' benefit, it is not known how often a reduced fee is given. It is also not known how many implant surgeons recycle used healing abutments from one patient to the next, but unless these materials can be adequately cleaned and sterilized, this practice should be reevaluated in light of the findings from this study for the following reasons: First, soft tissue integration is influenced by the material's characteristics. *In vitro* studies on animals and humans have confirmed the role of biocompatible oxide layer of titanium or its alloy to possess appropriate chemical composition that enhances both epithelial cells and connective tissue fibroblasts to adhere, spread, and proliferate. 5 8 12 23 Second, surface-free energy is seen to be high with a clean surface and conversely, and low where a contaminated surface exists. The higher the surface free energy the better the wettability of the surface with respect to cell attachment and spreading. Surface texture can also have a profound effect. It has been demonstrated that epithelium and human gingival fibroblasts attach and spread more readily on polished surfaces and that cells are sensitive to features as small as 0.2µm.

To have good interaction of the tissue and osseointegration, materials' biocompatibility and roughness of the surface play an important role. Based on a widely accepted categorization, implantable materials are classified into three distinct categories: biotolerant, bioinert, and bioactive. Biotolerant materials exhibit a healing mechanism known as “distant osteogenesis,” wherein the ions produced by the material when implanted into the host organism disrupt cellular metabolism and stimulate the formation of fibrous connective tissue. Bioinert materials do not discharge ions or hazardous compounds that may potentially impact cell metabolism or induce undesirable tissue reactions. Consequently, a phenomenon known as “contact osteogenesis” takes place, wherein connective tissue is not interposed. Bioactive compounds elicit a positive response by promoting bone deposition, thereby establishing chemical connections with tissue components such as hydroxyapatite, or by boosting cellular activity. 27 28 29 Goyal and Kaur observed increase in roughness resulted in the surge implant surface area, which indirectly resulted in better cellular attachment, and thus augment osseointegration. 30 Different sterilization methods have different modes of action. Autoclaving is a gold standard physical method of sterilization, which exposes the living organisms to unsustainable conditions of temperature, pressure, and time (121°C, 18 PSI, 20 minutes). However, dependent on the material density, volume, and size, the effectiveness of the autoclave may vary. 31 Chemical methods such as oxygen plasma are alternatives to conventional autoclaving, 32 in which ionized gas bombards the substratum surface and promotes the formation of free radicals under vacuum. 33 Active polar groups interrupt surface layers by stripping the surface layer, the thickness of which together with new properties are modified by characteristics of the gas used (type, purity, pressure, and position). 34

Surface roughness in this study was higher in both Genesis and Bredent group following the second autoclave cycle at 243.7 and 528.9 and the lowest in the control group with 79.0 and 157.6, which were significant at *p* -value less than 0.001. This is in agreement with the results obtained in a study by Park at al 31 who reported surface properties to be modified by both cleaning and sterilization on pretreated titanium (sandblasted and acid etched) and concluded that both hydrophobicity and roughness were not the same as that of the unused titanium surface. He further reiterated that such procedures also affected osteogenic differently (MG63 osteoblast) especially after autoclaving. Keller et al 35 also observed modification from 3 to 25 nm in the titanium oxide layer while in steam sterilization. They also stated that the modification in the surface color of these implants was due to this increase in thickness. Young 36 in earlier study had demonstrated that alteration in the thickness of the oxide layer from 259 to 700 Å had produced modification in the color of the surface of the metal. Increased surface roughness in the present study indicated changes on the surface resulting due to repeated sterilization of implant abutment. Vezeau et al 37 mentioned that multiple sterilizations of pure titanium implants can interfere in the bioacceptability of the material as well as cause alteration in the implant surface. Rough surfaces can accrue subgingival plaque (25 times) than their smooth counterpart, which is the basis of using smooth surfaces in long zygomatic implants. “Smooth” surfaces with roughness values smaller than the “critical threshold” of Sa = 0.2 μm (arithmetical mean height) are often preferred for abutments. 38 In a clinical setting, the implant–tooth connection for retention may be related to the abutment's bending capabilities; biomechanical factors must be considered. The objective of these approaches is to enhance the adaptability of bone in locations predominantly consisting of trabecular bone, such as the posterior maxilla. Currently, two primary approaches are being evaluated. Addition of biological mediators to the implant surface (such as cell adhesion or bioactive peptides, growth factors) or construction of reproducible nanoscale surface features are the two approaches. Surface engineering methods are also being developed to comprehend more about the characteristics, behavior, and reactions of various materials, which will allow for the future creation of novel materials, modification of existing procedures, and design of bioimplants. 39 40

## Strength, Limitations, and Bias

This study is one of the first studies which highlight that repeated sterilization after repeated adjustments can alter the surface characteristic of implant abutment and cause an increase in marginal gap. This study is limited by being an *in vitro* study and does not take into consideration the intraoral influences upon the implant abutment like presence of saliva and electrolytes. This study also did not include the effect of bacterial colonization before and after autoclaving that can be done in future studies. This study also investigated only two autoclaving cycles, while clinically there are more than two cycles of autoclaving that implant abutments are put through. This manuscript has been checked with the Fi-index tool and obtained a score of 0 for the first author only on the date 10/05/2023, according to SCOPUS. 41 42 The fi-index tool aims to ensure the quality of the reference list and limit any auto citations.

## Conclusion

With repeated use and modification required of implant abutments, sterilization treatment has certain effect on the margins and the surface properties of implant abutments. In our study, we found that marginal gap increased with autoclaving for both Genesis & Bredent group of implant abutment systems, although Bredent implant abutments showed consistent results. Surface roughness increased with autoclaving for both Genesis & Bredent group of implant abutment systems.
